# Shooting hoops: globetrotting plasmids spreading more than just antimicrobial resistance genes across One Health

**DOI:** 10.1099/mgen.0.000858

**Published:** 2022-08-12

**Authors:** Cian Smyth, Robert J. Leigh, Sarah Delaney, Richard A. Murphy, Fiona Walsh

**Affiliations:** ^1^​ Antimicrobial Resistance & Microbiome Research Group, Department of Biology, Maynooth University, Maynooth, Co. Kildare, Ireland; ^2^​ Alltech European Bioscience Centre, Dunboyne, Co. Meath, Ireland

**Keywords:** plasmids, antimicrobial resistance, food chain, virulence, colicin

## Abstract

Our study provides novel insights into the global nature of antimicrobial resistance (AMR) plasmids across the food chain. We provide compelling evidence of the globetrotting nature of AMR plasmids and the need for surveillance to sequence plasmids with a template of analyses for others to expand these data. The AMR plasmids analysed were detected in 63 countries and in samples from humans, animals and the environment. They contained a combination of known and novel AMR genes, metal resistance genes, virulence factors, phage and replicon types.

## Data Summary

The genomic data files are available under BioProject PRJNA816331. The publication title is also included in the bioproject details to aid discovery.

**Impact Statement**

Our study shows that it is not only the movement of antimicrobial resistance (AMR) genes across our food chain that are the same but the pieces of DNA carrying them are the same in chickens, food, soi, wastewater and human infections. This article adds detailed data to the field and highlights the need to look at the pieces of DNA carrying the AMR genes in addition to the AMR genes when investigating the movement of AMR across our food chain.

## Introduction

The World Health Organisation (WHO), Food and Agricultural Organisation of the United Nations (FAO) and World Organisation for Animal Health (OIE) identified antimicrobial resistance (AMR) as a critically important One Health problem. In 2017 WHO identified 12 global priority pathogens classified as containing important AMR, including plasmid-mediated carbapenem-resistant, extended spectrum beta-lactamase (ESBL)-producing or fluoroquinolone-resistant Gram-negative pathogens [1]. The European Union summary report on AMR in zoonotic and indicator bacteria from animals and food in 2018/19 frequently identified ampicillin-, sulfamethoxazole-, trimethoprim- and tetracycline-resistant *Escherichia coli* across pig, poultry and bovine populations [2]. *E. coli* with ‘very high’ resistance to ciprofloxacin and chloramphenicol were identified in poultry [2]. Due to the focus of many studies on *E. coli* we know much less about AMR in non-pathogenic and other zoonotic bacteria present in food-producing animals. There is a dearth of information on the AMR plasmids contained within the non-pathogenic or zoonotic bacteria within food-producing animals. To date, plasmid data have mainly been limited to plasmids present in culturable pathogens.

Plasmids enable AMR gene (ARG) transfer across bacteria and biomes and through the food chain. Thus it is these plasmids that are the risk to the further spread of AMR across One Health from animals to humans or the environment and vice versa. The contribution of specific plasmids to the dissemination of ARGs across One Health is unknown. Analysis of AMR plasmids conferring AMR or multi-drug resistance (MDR) may enable us to track and understand the dynamics of the spread of these mechanisms of resistance across One Health. Through comparison of the entire plasmids will we be able to track and trace their movements across One Health and identify their risk to One Health. Our study aimed to characterize the AMR plasmids isolated from poultry that could be expressed in *E. coli* in the context of other resistance and virulence genes and identify their global nature by comparison with all sequenced plasmids across One Health of human, animal and the environment.

## Methods

### Sample collections

Broiler caecal samples (*n*=34) were obtained from a commercial poultry production unit in the UK. The poultry were all fed the same diet. No antimicrobials were supplemented in their diet. All animals were taken from a commercial hatchery and transported to the commercial sheds on the day of hatching. Approximately 10 000 birds were mirror imaged from the hatchery into the production sheds. The birds were raised and fed under typical commercial production conditions, receiving feed and water *ad libitum*. All other conditions were kept uniform for all sheds. At days 21 (D21) and 34 (D34) post-hatch, the intact caecal pouches of randomly caught birds were removed immediately after euthanization. The entire contents of each caecal pouch were removed, lyophilized and stored at −80 °C until analysis.

### Exogenous plasmid isolation

Plasmids harbouring ARGs were isolated from each of the 34 caecal samples using the exogenous plasmid isolation method, as previously described [3]. Briefly, the total transferable plasmid populations from each of the ‘donor’ caecal samples (*n*=34) were individually transferred to the ‘recipient’ rifampicin-resistant *E. coli* DH5α via biparental mating. Exogenous transconjugants were selected on Eosin Methylene Blue (EMB) agar (Sigma) with rifampicin (100 mg l^−1^) and ampicillin, tetracycline, gentamicin, colistin, cefotaxime or ciprofloxacin at breakpoint concentrations according to CLSI guidelines [4]. From each antimicrobial selective plate with growth after exogenous isolation, a transconjugant was selected at random. If the same plate appeared to have bacteria with different features (colour, morphology), representatives of each colony type were selected.

### Antimicrobial susceptibility analysis

Antimicrobial susceptibility testing was performed on the exogenous transconjugants via the disc diffusion method according to CLSI guidelines for ampicillin, tetracycline, kanamycin, cefotaxime, ciprofloxacin, gentamicin, trimethoprim, imipenem and chloramphenicol antimicrobials [4]. The presence of ESBL enzymes was investigated using the CLSI confirmatory combination disc test method [4]. The test was considered positive when an increase in the growth-inhibitory zone around the cefotaxime disc with clavulanic acid was 5 mm or greater than the diameter around the disc containing cefotaxime alone.

### Plasmid sequencing

Plasmids from ESBL-positive transconjugants across D21 and D35 were selected for plasmid sequencing (*n*=9 exogenous transconjugant *E. coli *). The plasmids were extracted from the transconjugants using the Macherey-Nagel NucleoSpin Plasmid kit following the low-copy number protocol according to the manufacturer’s guidelines. Each transconjugant contained several plasmids, and thus 22 plasmids were sequenced to closed circular plasmids.

### Sequencing

Extracted plasmids were sequenced using the SQK-LSK-109 Ligation Sequencing kit [Oxford Nanopore Technologies (ONT)] with the NBD-104 Barcoding kit used for multiplexing. No departures from standard kit protocols were made. Completed libraries were run on an ONT MinION.

### Pre-assembly quality control (QC)

DNA outputs were basecalled and demultiplexed using ONT-Guppy v.1.1.alpha17-6-g5cecf99, nd this process was also used to remove any attached barcodes and adaptors. Filtlong v.0.2.0 was used to filter for any excessively short reads as well as filtering for any reads of low quality based on *qQ*-score [5].

### Assembly

Reads were *de novo* assembled using Unicycler v.0.4.9 long-read only [6]. This package was run with default parameters.

### Post-assembly QC

Visual assessment of assembled contigs was performed with Bandage v.0.8.1 [7]. This allowed for an easy inspection of circularized contigs and their size/depth.

### Plasmid annotation

In total, 22 plasmids were observed to be circular using Bandage. One other plasmid-derived sequence was observed to be both long (41 468 bp) and of high coverage. Each plasmid sequence was annotated using Prokka v.1.14.6 using the RNA profiling flag (--rfam) and, otherwise, default settings [8]. Due to the high level of hypothetical genes arising from Prokka annotation, each plasmid sequence was further annotated using BAKTA v.1.2.1 with default settings [9]. Annotations derived from BAKTA are provided as supplementary data [10]. Plasmids were visualized using in-house software. The sample name indicates the transconjugant and the second number the specific plasmid; for example, F4_7 is transconjugant 4 with plasmid number 7.

### Resistome, virulome and secondary metabolism profiling

The total plasmid DNA of each sample and each circularized plasmid was analysed for the presence of ARGs using ABRicate v.1.0.1 for AMR using the Comprehensive Antimicrobial Resistance Database (CARD) v.3.09 [11, 12]. Each circular plasmid was profiled in addition for metal and biocide resistance using BacMet v.2.0, and for virulence factors using the Virulence Factor Database (VFDB) v.5 [10, 13]. As plasmids often display rapid evolutionary rates, the minimum percentage identity (--minid) flag was reduced to 50 % (from the default 80%). As ABRicate requires a nucleotide input and as BACMET is only provided in amino acid format, the amino acid database was back translated (using translation table 11) prior to annotation [14]. As resistance may arise via point mutation, PointFinder v.3.1.1 was used with all available bacterial profiles (* Campylobacter* spp., *Enterococcus faecalis *, *Enterococcus faecium *, *Escherichia coli *, *Helicobacter pylori *, *Klebsiella* spp., *Mycobacterium tuberculosis *, *Neisseria gonorrhoeae *, *Salmonella* spp. and *Staphylococcus aureus *) to assess for resistance arising in chromosomally translocated genes [15]. Despite exhaustive searching, no resistance arising via point mutation was detected.

### Plasmid clustering and global relatedness

Each circular plasmid was searched against each other plasmid sequenced for this study using the distance function (dist) in Mash v.2.2.2 [16]. Instances where both the distance score (*D*) and *P*-value (*P*)≤0.1 were considered homologous. The 0.1 score filters were chosen to replicate the cut-offs used during the construction of PLSDB v.0.1.7 [17]. Each circular plasmid analysed in this study was assessed against all plasmids in PLSDB v.0.1.7 using the distance Mash dist algorithm. Again, instances where both the distance score (*D*) and *P*-value (*P*)≤0.1 were retained for further inspection. Each retained hit was further annotated using the metadata provided by PLSDB. The MASH distance score is highly correlated to the average nucleotide identity (ANI; a pairwise measure of genomic similarity between two genome’s coding regions subtracted from 1 (1−ANI) and a distance score of ≤0.05 equates to a ≥95 % ANI [16, 18]. A distance score of 0.1 is assumed to equate to ≥90 % ANI.

### Geographical mapping

Geocoordinates for each mapped plasmid were extracted from PLSDB metadata (where available) and converted to a representative country using the ‘Nomatim().reverse’ function in the GeoPy v.2.2.0 python library. The list of countries associated with each plasmid was used to annotate a world atlas to visualize the extent of spread.

### Temporal analysis of plasmid character state evolution

Where available, dates-of-isolation were extracted for each mapped plasmid from PLSDB. Each date-of-isolation was represented in year format to standardize the data, and plasmids without dates-of-isolation were excluded from further analyses. Drug resistance, metal resistance and virulence factor profiles were established for each plasmid using ABRicate as above, and metaprofiles were constructed for each plasmid group and ranked based on year (Supplementary Data, available with the online version of this article). As F5_3 (suspected phage-plasmid) only matched two other plasmids (and none was observed to contain a resistance gene or virulence factor), these plasmids were not subjected to further temporal analysis.

### Prophage detection

Each circular plasmid was assessed for prophages using Phigaro v.2.3.0 under default settings [19]. Detected prophages were individually annotated using BAKTA and assessed for resistance and virulence profiles using ABRicate with the CARD, BACMET and VFDB databases as above. A database of International Committee on the Taxonomy of Viruses (ICTV) exemplar virus genomes was downloaded from NCBI Assembly [20]. Each prophage was searched against the ICTV database using the Mash screen algorithm using instances where both identity ≥0.5 (50 %) and *P*≤0.1 are reported; hits from non-phage genomes were removed. The identity stringency score was lowered to 50 % to allow for detection of rapidly degrading or mutating phage fragments.

## Results

### Antimicrobial resistance profiles

Nine ESBL-positive transconjugant samples (Table 1) were resistant to ampicillin, cefotaxime and tetracycline. Transconjugants 3, 4, 5, 8 and 9 were also resistant to aminoglycosides (3, 8, 9), trimethoprim (4, 8, 9), ciprofloxacin (5, 9) or imipenem (8), or a combination.

**Table 1.** Antimicrobial resistance profiles and number of AMR genes within each ESBL-positive transconjugants

Sample	AMR profile	No. of genes per sample total DNA								
		Aminoglycosides	Bacitracin	Beta-lactamases	Lincosamides	QACs	Sulphonamides	Tetracycline	Trimethoprim	Total
1	AMP, CTX, TET	2	0	6	0	0	0	3	0	11
2	AMP, CTX, TET	0	0	1	2	1	1	2	0	7
3	AMP, CTX, KAN, CN, TET	0	2	3	0	0	0	2	2	9
4	AMP, CTX, TET, W	2	0	3	0	0	0	2	0	7
5	AMP, CTX, TET, CIP	3	0	1	0	2	1	1	1	9
6	AMP, CTX, TET	0	2	2	0	0	0	0	0	4
7	AMP, CTX, TET	2	0	4	0	0	0	2	0	8
8	AMP, CTX, KAN, TET, W, IMP	0	2	2	0	0	0	0	0	4
9	AMP, CTX, KAN, TET, CIP, W	3	0	5	0	0	2	3	3	16

AMP, ampicillin; CIP, ciprofloxacin; CN, gentamicin; CTX, cefotaxime; I, intermediate; IMP, imipenem; KAN, kanamycin; QACs, quaternary ammonium compounds; R, resistant; S, susceptible; according to CLSI guidelines (2018).; TET, tetracycline; W, trimethoprim.

### Plasmid descriptions

Both the total sequenced DNA (circularized and non-circularized) plasmid data and the circularized plasmids were analysed. The AMR genes detected across the total DNA (Tables 1 and 2, Supplementary data 1) conferred resistance to seven antimicrobial classes and quaternary ammonium compounds (QACs). However, some of the phenotypes and genotypes did not match. While *bla*TEM and *bla*SHV beta-lactamase genes were detected across all samples, only three contained the ESBL *bla*SHV-2 gene. Others contained blaSHV genes, which are not classified as beta-lactamases or ESBLs [21]. Two samples only contained the beta-lactamase *bla*TEM-1 genes. No mobile quinolone resistance genes were detected in the ciprofloxacin-resistant samples and nor were mutations in their quinolone resistance regions (QRDRs). Sample 6 contained no tetracycline resistance genes and sample 8 no imipenem or aminoglycoside resistance genes. Sample 4 was resistant to trimethoprim but contained no known resistance gene. Thus, as the phenotype was present, these isolates must contain novel resistance genes hidden within their plasmid DNA.

**Table 2.** Clustering groups, replicon types and ARGs detected in each circularized plasmid

Plasmid ID	Cluster group	Replicon type	Antimicrobial resistance genes												
			Total number of ARGs	*ANT(3'')-IIa*	*APH(3'')-Ib*	*APH(6)-Id*	*SHV-2*	*TEM-1*	*dfrA1*	*dfrA8*	*lnuG*	*sul1*	*tet(A*)	*tet(B*)	*dfrD*
F1_2	A	IncB/O/K/Z_2	1	.	.	.	Y	.	.	.	.	.	.	.	.
F4_2	A	IncB/O/K/Z_2	1	.	.	.	Y	.	.	.	.	.	.	.	.
F6_1	A	IncB/O/K/Z_2	1	.	.	.	Y	.	.	.	.	.	.	.	.
F7_2	A	IncB/O/K/Z_2	1	.	.	.	Y	.	.	.	.	.	.	.	Y
F9_3	A	IncB/O/K/Z_2	1	.	.	.	Y	.	.	.	.	.	.	.	.
F1_17	B	Col440II_1 and ColRNAI_1	0	.	.	.	.	.	.	.	.	.	.	.	.
F4_7	B	Col440II_1 and ColRNAI_1	0	.	.	.	.	.	.	.	.	.	.	.	.
F7_6	B	Col440II_1 and ColRNAI_1	0	.	.	.	.	.	.	.	.	.	.	.	.
F9_5	B	Col440II_1 and ColRNAI_1	0	.	.	.	.	.	.	.	.	.	.	.	.
F3_4	C	IncN_1	2	.	.	.	.	Y	.	.	.	.	Y	.	.
F5_2	C	IncN_1	2	.	.	.	.	Y	.	.	.	.	Y	.	.
F8_22	C	IncN_1	2	.	.	.	Y	.	.	.	.	.	Y	.	.
F1_1	D	IncFIB_1	4	.	Y	Y	.	Y	.	.	.	.	.	Y	.
F2_2	D	IncFIA_1, IncFIB_1, IncFIC(FII)_1	1	.	.	.	.	Y	.	.	.	.	.	.	.
F4_1	D	IncFIB_1	4	.	Y	Y	.	Y	.	.	.	.	.	Y	.
F5_1	D	IncFIB_1	7	Y	Y	Y	.	Y	Y	.	.	Y	Y	.	.
F9_1	D	IncFIB_1	4	.	Y	Y	.	Y	.	.	.	.	.	Y	.
F1_18	Singleton	Col440II_1	0	.	.	.	.	.	.	.	.	.	.	.	.
F1_3	Singleton	IncFII(pECLA)_1, IncI1_1_Alpha	1	.	.	.	.	Y	.	.	.	.	.	.	.
F2_1	Singleton	IncFIA(HI1)_1_HI1, IncHI1A_1, IncHI1B(R27)_1_R27	4	Y	.	.	.	.	.	.	Y	Y	.	Y	.
F3_6	Singleton	IncX1_1, IncX3_1	2	.	.	.	.	Y	.	Y	.	.	.	.	.
F5_3	Singleton	None	0	.	.	.	.	.	.	.	.	.	.	.	.
F9_2	Singleton	IncFIB(pHCM2)_1_pHCM2	0	.	.	.	.	.	.	.	.	.	.	.	.

### Circularized plasmid descriptions

We resolved 22 circularized plasmids and one linear plasmid with a very high coverage but which could not be circularized (sample 8). The circularized plasmid sizes were highly variable (3.365–190.3 kb); five plasmids were less than 10 kb and 12 exceeded 100 kb (Supplementary Data 2). Coding proportions were stable across all samples except for plasmid F4_7, which displayed a considerably lower coding proportion (0.585). Variation in GC% variation was observed (0.448–0.552). Sixteen plasmids contained an *oriC*, and both F2_2 and F3_6 contained three *oriC* copies. Comparatively, 19 plasmids contained an *oriT*. The plasmids F2_1, F5_3 and F9_2 lacked both an *oriT* and *oriC*.

### Plasmid clustering of circularized plasmids

Circularized plasmids (*n*=22) plus plasmid 8_22 clustered into one of four groups (A–D) or were singletons (Table 2, Supplementary Data 2). Group A comprised five plasmids (F1_2, F4_2, F6_1, F7_2 and F9_3); Group B four plasmids (F1_17, F4_7, F7_6 and F9_5); Group C three plasmids (F3_4, F5_2 and F8_22); and Group D five plasmids (F1_1, F2_2, F4_1, F5_1 and F9_1). All other plasmids were singletons (*n*=5). A range of replicons were present across the plasmids (Supplementary Data 3). Replicon types were generally consistant within cluster groups: Group A: IncB/O/K/Z_2, Group B: Col440II_1 and ColRNAI_1, Group C: IncN_1 and Group D: IncFIB – within this group plasmid F2_2 also contained IncFIA and IncFIC replicons. The remaining six plasmids contained Col440II_1 (F1_18), IncFII and IncI1 (F1_3), IncFIA, IncHIA and IncHIB (F2_1), IncX1 and IncX3 (F3_6), no replicon type (F5_3), and FIB (F9_2). Col440II_1 and ColRNAI_1 plasmids were less than 7 kb. The plasmids between 40 and 50 kb were either IncN or IncW and those greater than 100 kb were IncB/O/K/2, IncF or IncH. This demonstrates the wide range of plasmid types identified across the samples and with similar AMR profiles. There was no correlation between replicon type and resistance phenotype or genotype.

### Resistance profiling of circularized plasmids

Beta-lactam resistance genes were identified in 15 of 23 plasmids, ten of which (all Group D, C, and singleton plasmids F1_3, F3_4 and F3_6) contained the penicillinase *bla*TEM, and five (all Group A) contained ESBL *bla*SHV-2 (Supplementary Data 4). No plasmid contained both *bla*TEM and *bla*SHV. Tetracycline-resistant genotypes were observed in plasmids F5_1 via *tetA* alone, both *tetA* and *tetB* in F1_1, and in F2_1, F4_1 and F9_1 via *tetB* and *tetC*. The tetracycline resistance repressor gene *tetR(B*) and the *tetC* genes were detected beside the *tetB* genes and the *tetR(A*) gene beside the *tetA* gene in F5_1. Plasmid F1_1 contained only the *tetR(B*) gene. Plasmids F5_2 and F8_22 contained the *tet*(C) and *tetR(A*) genes and F3_4 contained only the *tetR(A*) repressor. Aminoglycoside resistance genes *aph(3’’)-Ib* and *aph(6)-Id* conferring streptomycin resistance were observed together (F1_1, F4_1, F5_1, F9_1). Plasmid F2_1 contained *aadA22* and F5_1 also contained *aadA1*, both of which confer resistance to streptomycin and spectinomycin. Three singleton plasmids displayed trimethoprim resistance genotypes: F3_6 via *dfrA8,* F5_1 via *dfrA1* and F9_2 via *dfrD*. However, neither of the *dfrA*-containing transconjugants displayed resistance to trimethoprim. Fourteen sequential point mutations were observed between nucleotides 133 and 146, followed by an 11 amino acid deletion event from sites 147–157 in *dfrA1*. These mutations are at the C terminus of the Dfr Pfam domain (PF00186) and, while the active site is at the N terminus of this domain, the mutations may limit binding potential. No point mutations were detected in *dfrA8*. Transposon-mediated lincosamide resistance (*lnuG*) was observed in F2_1. Sulphonamide resistance *sul1* genes were observed in plasmids: F2_1 (singleton) and F5_1 (Group D). Both were adjacent to the QACs efflux SMR transporter *qacEΔ1*. No plasmid-mediated carbapenemase or colistin resistance genes were detected, and thus these plasmids did not confer resistance to the last line of defence antimicrobials. A multidrug (aminoglycoside, β-lactam and tetracycline) resistance genotype (*via aph(3’’)-Ib*, *aph(6)-Id, bla*TEM and *tetA*) was observed in four Group D plasmids. The exception was plasmid F2_2, which contained only *bla*TEM. Plasmid F5_1 (Group D) additionally contained sulphonamide and trimethoprim resistance genes. Group B plasmids, F1_17, F4_7, F7_6, F9_5 and singletons F1_18 and F5_3 contained no AMR, metal resistance nor virulence genes.

### Metal resistance and virulence genes

Metal resistance gene (MRG) abundances varied across plasmids from zero (*n*=17) to two (F2_1), three (Group D F2_2), four (Group D F1_1, F4_1, F9_1) or 11 (Group D F5_1) (Supplementary Data 5 and 6). Each plasmid with MRGs also contained ARGs. The Group D plasmids comprised the *sitABC* MRGs conferring resistance towards manganese, iron and hydrogen peroxide with the addition of the *merR* gene or, in the case of plasmid F5_1, all eight mercury resistance genes (*merABCDEPRT*) and *qacEΔ1*. F2_1 contained *corA,* which confers nickel, cobalt and manganese resistance, and *qacEΔ1*.

The MRG-positive plasmids also contained virulence factor genes: Group D plasmids (F1_1, F2_2, F4_1 F5_1 and F9_1) and the singleton F2_1. Plasmid F2_1 only contained the *astA* gene, the genotype for heat-stable enterotoxin 1 production. The Group D plasmids all contained the siderophore (salmochelin) production genotypes *via* the *iroBCDEN* cassette and displayed mammalian cell autophagy and phagosome escape protein *icsA/sopA*. The *iss* gene was identified across all Group D plasmids and the *cvaC* gene (colicin V) was detected in F2_2 and F5_1. The *iss* gene confers increased serum survival, which is important in protecting against phagocytosis. Seventeen plasmids contained no virulence factor genes.

### Phage identification

An unidentifiable yet transposable prophage was detected on three Group D plasmids (F1_1, F2_2 and F4_1) (Supplementary Data 7 and 8). Three *Myoviridiae* phages were observed on three singleton plasmids (F2_1, F5_3 and F9_2). Plasmid F9_2 also contained two individual *Siphoviridiae* prophages. Containment analyses suggested that all prophages were related to *Escherichia–Shigella–Salmonella* (ESS) clade phages (Supplementary Data 9), specifically an *stx*-converting phage. Further inspection of the unidentifiable phages revealed they contained no structural component genes and consisted almost entirely of transposition elements and regulatory machinery, which may indicate a novel intragenomic phage lifecycle (similar to a permanent lysogenic lifecycle without ever entering into the lytic cycle). The phage on F2_2 contained the macrolide resistance genes *macA* and *macB*. Further inspection of the *Myoviridae* prophages in plasmid F5_3 suggest intact prophages with full lytic enzymatic profiles (presence of holin, endolysin and spanin). The *Myoviridae* phage in F5_3 encompasses 84.38 % of the entire plasmid, suggesting that this plasmid may, in fact, be a circular phage genome or a newly described phage-plasmid [22].

### Global plasmid relatedness

The circularized plasmids were compared with all plasmids in PLSDB v.0.1.7 (*n*=34 513 plasmids) [23]. Most hits were observed to be within *Enterobacteriaceae* (Supplementary Data 10). *E. coli* was the most commonly associated host species, except for plasmid F1_18, where *Salmonella enterica* (*n*=29) was most prevalent but *E. coli* was next most prevalent (*n*=26). When metadata was complete, 15 plasmids (encompassing all of Groups A, C and D, and plasmids F5_3 and F9_2) were most commonly associated with plasmids isolated from human samples (Supplementary Data 10). Seven plasmids (encompassing all of Group B and plasmids F1_18 and F9_5) were most often associated with faecal samples from farm floors, five with pig faeces (F1_17, F1_18, F3_6, F4_7, F7_6), and one each with cow faeces (F7_6) and sheep faeces (F9_5). One plasmid was most commonly associated with river samples (F1_3). All plasmids, except plasmid F5_3, were associated with bird samples (Supplementary Data 10). Plasmid F5_3 was only associated with two human samples, a *bla*NDM-5 plasmid from *E. coli* (NZ_AP023208.1), isolated from a Japanese patient with no history of foreign travel, and the other from an *Escherichia albertii* isolated in the USA (NZ_CP024288) [24].

**Fig. 1.**
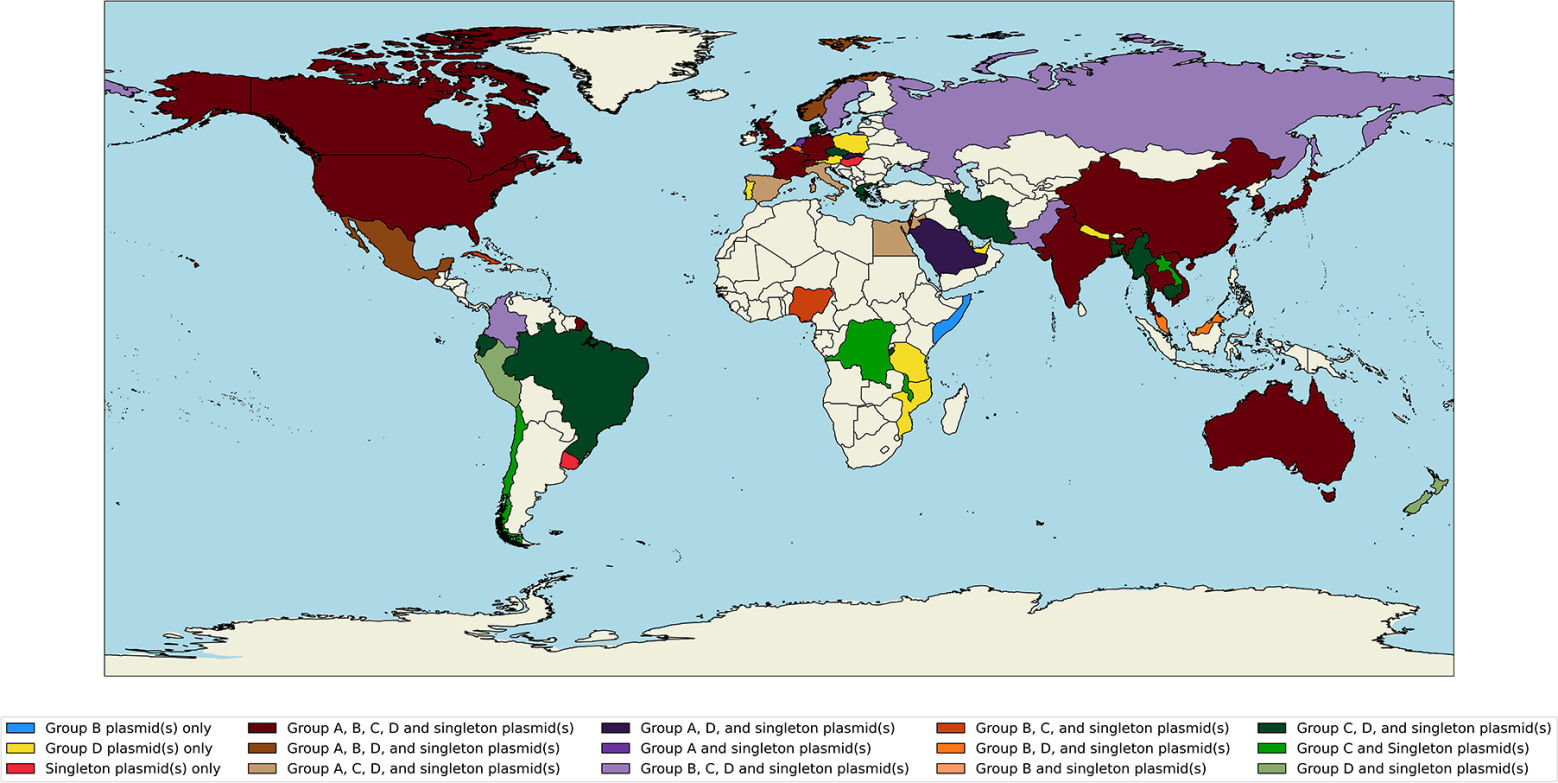
Global distribution world map of countries containing a plasmid from PLSDB associated with each plasmid extracted from the chicken samples.

The list of countries associated with each mapped plasmid extracted from PLSDB metadata was used to annotate a world atlas to visualize the extent of spread (Fig. 1a–e). In total, 63 countries were represented across all non-Antarctic continents. Each plasmid was observed in both the USA and Japan. All plasmids except F5_3 were observed in Canada, China and the UK. These data, as with all genomic data, are biased to include only the countries with data and metadata submitted to online databases. Thus, a lack of observation in a specific country does not necessarily equate to a lack of plasmid. We suggest that this is the first sighting of plasmid F5_3 in Europe and the global dissemination of the other plasmids.

### Temporal analysis of plasmid character state evolution

Drug resistance genotypes seem to be temporally linked, with most prominently used antibiotics correlating with resistance genotypes for given years [25]. Plasmids isolated between 1960 and 1979 were observed to confer resistance to aminoglycosides *[ANT(3'')-IIa, APH(3'')-Ib, APH(3')-Ia* and *APH(3'')-IIa*], β-lactams (*bla*OXA-2*, bla*TEM-1*, bla*TEM-150), phenicols (via *catI*), sulfonamides (*sul1* and *sul2*) and tetracyclines [*tet(A*) and *tet(B*)]. Comparatively, non-OXA-mediated ESBL resistance (*bla*CTX-M) was not observed until 2001, mirroring previous resistance profile reports.

The mercury resistance *mer* operon was the most prevalent MRG present across time. Resistance to silver (*sil* operon), to tellurite (*ter* operon) and to QACs were also well represented. Resistance to these heavy metals may also confer secondary oxidative stress mitigation [26].

## Discussion

AMR surveillance to date has predominantly focused on culturable pathogens, monitoring their phenotypic resistance to a range of antimicrobials and the identification of the genes. Plasmids mediate AMR and MDR gene transfer across bacteria, commensals or pathogenic bacteria of humans, animals and the environment [27]. However, due to difficulties in analysing plasmids most genomic studies have focused on genes or whole genome sequences rather than resolving the plasmid sequences. Our study identified a definitive spread of AMR plasmids across One Health as they all mapped to plasmids identified from human, animal or environmental samples. The lowest coverage of the One Health triangle identified was the environment. However, the number of complete plasmids sequenced from environmental samples is relatively low. This is a large gap in our knowledge as there are few data on the AMR plasmid content of water, soil or plant samples.

Our study demonstrates that the same plasmids have already transferred globally across 63 different countries, as demonstrated by the mapping of the plasmids based on the metadata. This study demonstrates that it is not only the AMR genes or the host pathogens moving across One Health but it is entire plasmids. There was no one specific biomarker that could group the isolated plasmids. There were different AMR genes, different replicon types, and variation across metal resistance and virulence factors. Further studies of plasmids with differing resistance profiles can use our template to explore the global and One Health nature of sequenced plasmids. This will provide novel insight into the global nature of every sequenced plasmid in the future.

The plasmids analysed in this study provide novel insight into the AMR, virulence and metal resistance plasmids present. While the AMR phenotype of the transconjugants included resistance to ciprofloxacin and imipenem, no known ARGs were detected. Thus, these plasmids are a reservoir of novel fluoroquinolone and carbapenem resistance genes. Sequenced plasmids contain a large number of uncharacterized hypothetical genes. Genes conferring resistance to a range of antimicrobials were also identified on the same plasmid. Additionally, multiple plasmids conferring resistance to one or two antimicrobials were detected in the same sample. There are many studies of the ESBL profiles and known ARGs conferring resistance in *E. coli* globally across One Health but relatively little information about the plasmids conferring ESBL resistance or other AMR in food animals and their link to those identified in humans, other animals or the environment [2]. Comparison of our plasmid sequences with those globally identified their presence in *E. coli* and other pathogens isolated from humans, other food animals and the environment across time. We identified plasmids lacking AMR, virulence genes or MRGs, which were all colicin E-type plasmids, suggesting the colicin E plasmids were transferred with AMR plasmids. Therefore, the use of antimicrobials may be also selecting colicin plasmids. Colicin has long been known as a plasmid-borne bacteriocin that kills other *E. coli* cells lacking the same plasmid [28].

Metal resistance and virulence genes associated with human extraintestinal pathogenic *E. coli* (ExPEC) and avian pathogenic *E. coli* (APEC) were present in Group D plasmids and sample F2_1 (*astA*). The virulence factors of APEC include *hlyE*, *cvaC*, *iss*, *fimC*, *tsh*, *lucC* and *sitA* [29]. However, there is no definitive list of virulence genes common to APEC and while it is the agent of coliobacilliosis it is also a commensal of the poultry gut. The plasmids identified contained only *iss*, *sitA* and *cvaC* (*n*=2), from the APEC virulence genes. Both *iss* and *cvaC* are important virulence factors in neonatal meningitis *E. coli* (NMEC), which are not present in uropathogenic *E. coli* but are present in APEC. However, they contained the core ExPEC genes present in NMEC: *iroBCDEN*, *icsA*/*sopA*, *sitABCD *and *hlyF* (*n*=4), one contained *iucABCD* and *iutA*, but none contained *ompT* nor *bor*. While the virulence factors do not definitively describe the plasmid as conferring virulence in a specific ExPEC, the genes present display the potential to confer virulence to hosts including poultry and humans.

Temporal data sampling was strongly biased towards the past decade, probably due to falling sequencing costs allowing for more frequent analyses, and strongly biased towards *Enterobacteriaceae *, specifically the genera *Escherichia, Salmonella* and *Shigella *. A general trend was observed across all plasmid groups whereby different resistance genes and virulence factors were transiently incorporated and lost over time, suggesting differential evolutionary pressures; however, as the plasmid network is non-transitively retained, these results suggest a highly successful, yet malleable and dynamic, plasmid backbone (Supplementary Data 13–20). Due to the aforementioned bias, the diversity and malleability of these plasmids is most noticeable is samples isolated in the past decade. Despite the global persistence of most plasmid groups, neither resistance nor virulent genotypes seem to be largely geographically enriched.

Three of four plasmid groups (Groups A, C and D) were associated with clinical isolates, and Group B was most commonly associated with agriculture, although all plasmids (except F5_3) were also associated with food products. We detected β-lactam and tetracycline resistance across all samples. These are commonly observed in agricultural isolates so this result is consistent with current knowledge [30]. The presence of Ig domain-like genes within the F9_2 *Siphoviridae* phage suggests an important role in virulence. These proteins function as invasins and adhesins in both enteropathogenic *E. coli* (EPEC) and enterohaemorrhagic *E. coli* (EHEC) promoting the development of diarrhoeal disease [31].

## Conclusions

Our study provides novel insights into the global nature of resistance, virulence and colicin plasmids across the food chain and time. We have provided compelling evidence of the globetrotting nature of AMR plasmids and the need for surveillance to sequence plasmids using our template of analyses for others to expand these data. The AMR plasmids analysed may contain novel ARGs, which remain to be characterized. The analysis of AMR needs to include the investigation of plasmids globally to truly identify the risk to animal, human and environmental health from AMR and potential for co-selection by the use of QACs or metals.

## Supplementary Data

Supplementary material 1
